# Insights Into Exosomal Non-Coding RNAs Sorting Mechanism and Clinical Application

**DOI:** 10.3389/fonc.2021.664904

**Published:** 2021-04-27

**Authors:** Yi Qiu, Peiyao Li, Zuping Zhang, Minghua Wu

**Affiliations:** ^1^ Hunan Cancer Hospital and the Affiliated Cancer Hospital of Xiangya School of Medicine, Central South University, Changsha, China; ^2^ Cancer Research Institute, School of Basic Medical Science, Central South University, Changsha, China; ^3^ Key Laboratory of Carcinogenesis and Cancer Invasion of Ministry of Education, China National Health Commission Key Laboratory of Carcinogenesis, Xiangya Hospital, Central South University, Changsha, China

**Keywords:** exosome, extracellular vesicles, non-coding RNAs, sorting mechanism, cell-cell communication, ncRNAs

## Abstract

Exosomes are natural nanoscale bilayer phospholipid vesicles that can be secreted by almost all types of cells and are detected in almost all types of body fluids. Exosomes are effective mediators of cell–cell signaling communication because of their ability to carry and transfer a variety of bioactive molecules, including non-coding RNAs. Non-coding RNAs have also been found to exert strong effects on a variety of biological processes, including tumorigenesis. Many researchers have established that exosomes encapsulate bioactive non-coding RNAs that alter the biological phenotype of specific target cells in an autocrine or a paracrine manner. However, the mechanism by which the producer cells package non-coding RNAs into exosomes is not well understood. This review focuses on the current research on exosomal non-coding RNAs, including the biogenesis of exosomes, the possible mechanism of sorting non-coding RNAs, their biological functions, and their potential for clinical application in the future.

## Introduction

Since the discovery of extracellular vesicles (EVs), researchers have found EVs in almost all biological fluids. EVs have received unprecedented attention owing to their key roles, such as their potential as biomarkers for liquid biopsies and therapeutic applications (1, 2). Based on their formation, typical EVs are roughly divided into three categories: microvesicles (also called outer membrane vesicles, ectosomes, or shedding vesicles), exosomes, and apoptotic bodies. Microvesicles (100–1000-nm diameter) are released from the cell membrane through the blebbing and fission of the plasma membrane. Apoptotic bodies (50–5000-nm diameter) fall off during the process of cell apoptosis (3–6). Exosomes derived from the endosomal compartment are phospholipid bilayer vesicles with a diameter of 50–150 nm, generated by sequential invagination of the plasma membrane and endomembrane (7–9). They are specifically loaded with cargos, depending on the producer cell type and its homeostatic state. This review focuses on exosomes, which are the most studied type of EVs. Exosomes contain a variety of bioactive substances, including DNA, RNA, proteins, and lipids, which are secreted into the extracellular space and absorbed by target cells to change their phenotype. Functional exosomes can protect packaged cargos from being destroyed by various biological enzymes, extending the circulating half-life and enhancing exosomal RNA biological effects (10).

Accumulating evidence suggests that most pluricellular organisms sustain exosome-based communications *via* the intercellular exchange of non-coding RNAs (ncRNAs) between cells. Valadi et al. first reported exosomes containing mRNAs and microRNAs (miRNAs) (11). Subsequently, a large number of studies have confirmed the existence of many other ncRNA species in exosomes (12–15). More than 98% of the human genome is composed of ncRNAs that are involved in a broad range of physiological or pathophysiological processes in humans (16). NcRNAs play an indispensable role in genetic expression, epigenetic regulation, RNA splicing and translation processes, protein degradation, and transport, among others. Exosomal ncRNAs, especially those derived from tumors, are highly enriched and stable and act as messengers in cell–cell communications (17, 18). This review mainly focuses on three types of ncRNAs: miRNAs (average length of 22 nucleotides), long ncRNA (lncRNA) (>200 nucleotides in length), and circular RNA (circular molecule that has a covalently closed loop structure, lacking a poly A tail or 5′→3′ polarity) (19–21).

To advance the field of exosomal biology and understand the roles of exosomal ncRNAs, this review focuses on the cellular machinery and processes of exosome formation, sorting of ncRNAs into the exosome pathway, and biological roles of the three types of exosomal ncRNAs. The application of exosomal ncRNAs as biomarkers and the potential roles of exosomal ncRNAs as therapeutic agents are also discussed.

## Biogenesis of Exosomes

**Figure 1:**
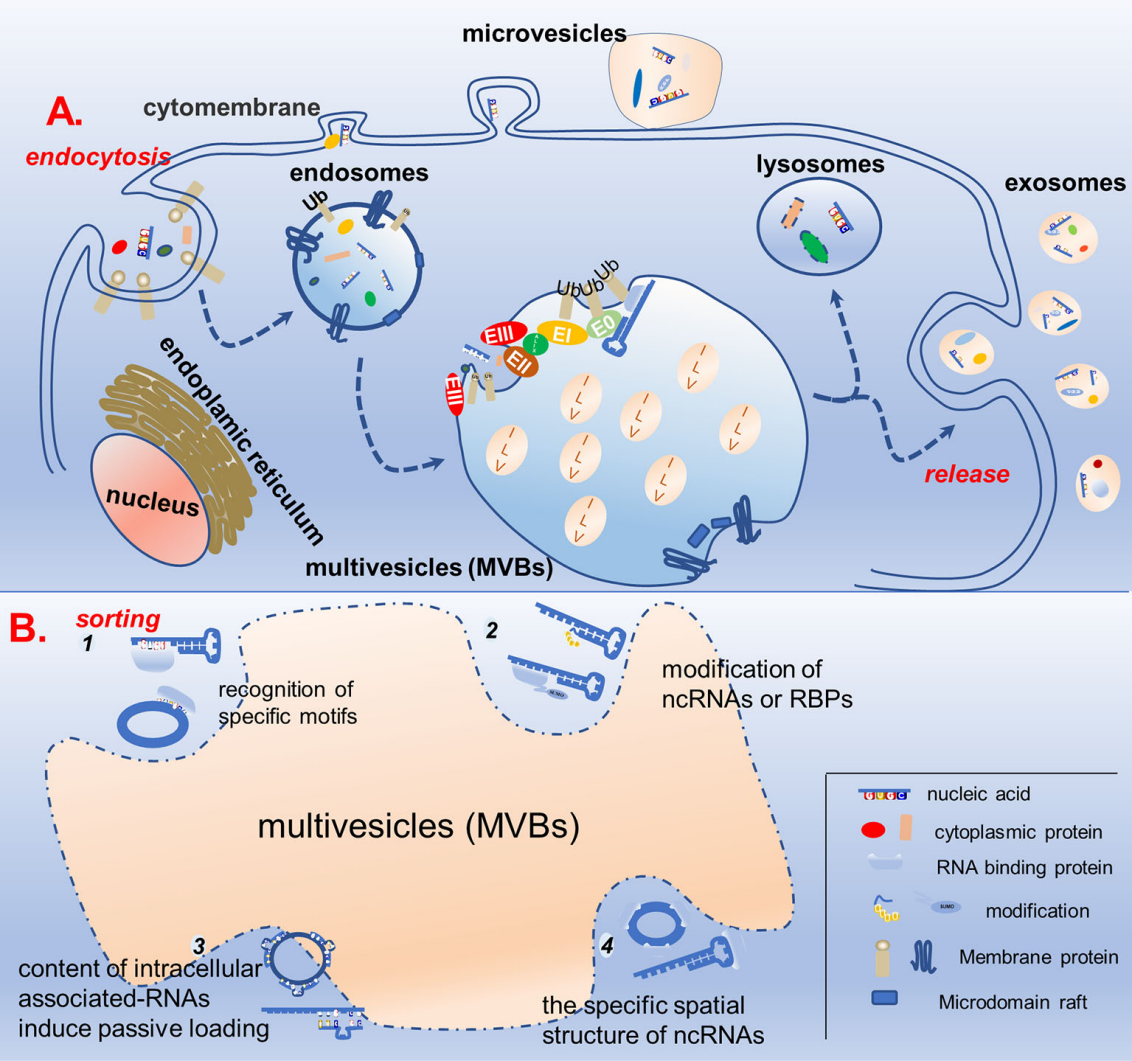
Schematic diagram of exosome biogenesis and potential mechanisms for sorting non-coding RNA. **(A)** Proteins and nucleic acids are secreted into extracellular vesicles through budding and fission of the cell membrane, while others enter the cell through the endocytosis pathway to form endosomes, which further mature into multivesicles bodies. In the multivesicles, membrane invagination, budding, and fission results in the formation of ILVs in ESCRT-dependent and ESCRT-independent manners. ILVs either fuse with lysosomes to degrade the cargos or dock with the cell membrane to be released, forming biological exosomes. **(B)** In the process of ILVs formation, specific non-coding RNAs are specifically sorted into ILVs. There are four possible mechanisms, as follows: (1) recognition of specific motifs on non-coding RNAs by RBP; (2) modification of non-coding RNAs or RBPs capable of binding to them to help them wrapped in ILVs; (3) the content of intracellular associated-RNAs modulate ncRNAs’ sorting into ILV; (4) the specific spatial structure of non-coding RNAs also affects its ability to enter ILVs.

Exosome biogenesis is closely related to the cellular endocytosis pathway. Endocytosis of the plasma membrane leads to the formation of early endosomes, which then bud inward and mature into late endosomes (LE). In turn, LEs develop into multivesicular bodies (MVBs) containing intraluminal vesicles (ILVs). Most MVBs fuse with lysosomes to degrade the contained cargo, whereas some bud and divide from the endosomal membrane to produce ILVs, which are released into the extracellular space when the endosomal membrane fuses with the cytoplasmic membrane, namely exosomes (22, 23). The biogenesis of exosomes has often been described to involve an endosomal sorting complex required for transport (ESCRT)-dependent or ESCRT-independent mechanism, although the pathways may not be entirely separated. In fact, the pathways may work synergistically, and different subpopulations of exosomes could depend on different machineries (**Figure 1**) (24–26).

### ESCRT-Dependent Mechanism in the Biogenesis of Exosomes

The ESCRT pathway was initially defined in yeast genetic screens to identify the factors necessary to sort membrane proteins into intraluminal endosomal vesicles. ESCRT-dependent exosome biogenesis includes ubiquitin-dependent and ubiquitin-independent ESCRT sorting signals. The canonical mechanism by which ESCRT is activated is initiated by the recognition of ubiquitinated membrane proteins on cell membranes. Ubiquitinated endosomal proteins that are deposited into the lumens of MVBs are either sorted for lysosomal-mediated degradation or secreted as exosomes into the extracellular milieu. The ESCRT system includes four protein complexes: ESCRT-0, ESCRT-I, ESCRT-II, and ESCRT-III (26, 27). The ESCRT-0 complex is first activated by phosphatidylinositol 3-phosphate PI(3)P and ubiquitinated molecules on the multivesicular membrane (28). Subsequently, the ESCRT-I complex is recruited by ESCRT-0 with the help of the hepatocyte growth factor-regulated tyrosine kinase substrate prosaposin (HRS PSAP) subunits, which can be promoted by heparanase (endosomal enzyme) (29). The interaction between ESCRT-I and ESCRT-II is mediated through VPS28 and EAP45 (Vps36) subunits, which are essential for sorting cargo in the MVBs and deforming the membrane, resulting in bud formation (30–32). ESCRT-III aggregates at the neck of the bud and causes the sprout to shear, thereby releasing ILVs and driving vesicle scission from MVBs. The VPS4/VTAL complex is hydrolyzed by ATPase, which provides energy to depolymerize the polymerized ESCRT-III for recycling to complete further budding processes (33). The membrane protein syntenin combines with the cytoplasmic protein syndecan and ALIX accessory protein to form a ternary complex. This complex can interact with the TSG101 domain of ESCRT I and the CHMP4 domain of ESCRT III and participate in the ESCRT-dependent pathway for exosome secretion (34–36).

Other groups of researchers recently reported that some ESCRT-related proteins can be recruited independently of ubiquitination to promote exosome secretion by cargos. The post-translational attachment of small ubiquitin-like modifier (SUMO) to proteins plays a vital role in mediating ESCRT-dependent sorting into EVs. SUMO is a small ubiquitin-like protein consisting of more than 100 amino acid residues with a molecular weight of approximately 12 kDa. It is named after ubiquitin, with which it shares a similar mode of action and spatial structure. SUMOylation is a covalent modification similar to ubiquitination in which the terminal diglycine configuration of SUMO forms an isopeptide bond with the E-amino group of the substrate protein lysine. Unlike ubiquitination, which often mediates the degradation of target proteins, SUMOylation modification is thought to enhance the stability of target proteins (37, 38). SUMO is recruited to ESCRT formation sites by interacting with phosphoinositols and requires the ESCRT subunits Tsg101 and VPS4, as well as the ESCRT-associated protein Alix. Furthermore, the release of the cytosolic protein α-synuclein within EVs in human cerebrospinal fluid is SUMO-dependent (39, 40). Liu et al. demonstrated the existence of a ubiquitin-independent pathway; for example, the COP9 signalosome (CSN)-associated protein CSN5 can regulate the sorting of exosomal proteins (such as HSP70 and HIV Gag protein) in both a deubiquitinating activity-dependent and -independent manner (41).

### ESCRT-Independent Mechanism in the Biogenesis of Exosomes

Katarina et al. were the first to discover an ESCRT-independent exosome formation mechanism. They provided evidence that intracellular ceramide was deposited with the depletion of the neutral sphingomyelinase enzyme, which induces the coalescence of small raft-based microdomains into larger domains, promoting domain-induced budding (42). Subsequently, Aude et al. revealed the presence of lipid raft microdomains in exosomal membranes and indicated their possible involvement in vesicle formation and structure (43). Tetraspanins, a family of four transmembrane proteins, were shown to be responsible for exosome formation (44). Similarly, Van Niel found that tetraspanin CD63 directly participates in ESCRT-independent biogenesis of the PMEL (a component of melanocyte lysosome-related organelles) luminal domain, rather than traditional ESCRT-dependent cargoes, to ILVs (45). CD82 and CD9, which are also tetraspanin membrane proteins, induce beta-catenin export *via* exosomes, which are blocked by a sphingomyelinase inhibitor (46). Other tetraspanin membrane proteins, such as Tspan8, including CD106 and CD49d, as well as CD81 (RacGTPase), are also considered to be involved in the ESCRT-independent pathway (47, 48). Exosomal cholesterol secretion depends on the presence of flotillin protein (49). Many other molecules are also involved in the formation of exosomes, and further details need to be explored (50–52).

Another ESCRT-independent mechanism is RAB GTPase-dependent exosome generation. RAB GTPases are positioned on the surface of a specific membrane structure and regulate the vesicle transport of the corresponding membrane structure by recruiting effector factors. For example, RAB5 regulates the formation and mutual fusion of the endoplasmic reticulum. The transition from RAB5 to RAB7 on the endosome membrane regulates the transition from the early endosome to the late endosome. RAB7 regulates the fusion of late endosomes and lysosomes to degrade ILVs, whereas RAB27 regulates the docking and fusion of MVBs and membranes to release ILVs to form exosomes. The membrane proteins of endocytosis, especially the receptor tyrosine kinase family epidermal growth factor receptor (EGFR), are located in the endosome and MVBs and initiate lysosomal degradation through the fusion of MVBs and lysosomes. Kang et al. conducted experiments to knock down the ESCRT components HRS and TSG101 as well as the related protein Alix. As a result, they found that these were not involved in the formation of EGFR exosomes driven by RAB31^Q65L^. Active RAB31 drove EGFR into MVBs to form ILVs and exosomes, whereas EGFR phosphorylated RAB31 to drive homologous exosomes. Flotillin protein in the lipid raft microdomain was involved in the formation of ILVs driven by active RAB31, which was independent of the ESCRT mechanism. It was further proved that RAB31 recruited TBC1D2B to inactivate RAB7, inhibit the fusion of MVBs and lysosomes, and further promote the production of exosomes (53–55).

## Possible Sorting Mechanism of Exosomal ncRNAs

The higher enrichment of certain ncRNAs in exosomes secreted by cells in specific states indicates that exosomal ncRNA encapsulation is an intense biological process that initiates exosomal ncRNA signaling. However, the exact cellular process responsible for selective specific exosomal ncRNA enrichment has not been well established in eukaryotic cells (**Table 1**).

**Table 1:** Summary of non-coding RNAs and their possible sorting mechanism.

RNA Type	Disease/source	Molecular partner	Sorting mechanism	Reference
miRNA	Endothelial cell	RBP: hnRNPU	Exosomal miR-30c-5p is selected through its motif AAMRUGCU binding to hnRNPU.	(56)
miRNA	Inflammation	RBP: FMR1	FMR1 and lysosomal protein cRILP co-ordinate the loading of miRNAs with AAUGC motif into exosomes.	(57)
miRNA	Cutaneous injury	RBP: hnRNPA2B1	SUMOylated hnRNPA2B1 directs the loading of certain miRNAs through the recognition of specific short motifs, such as the GGAG tetraloop.	(58, 59)
miRNA	Epithelial cells	RBP: hnRNPA2B1	Membrane protein cav-1 tyrosine 14 (Y14) phosphorylation interacts with the O-GlcNAcylated hnRNPA2B1, leading to a change in miRNA-17/93 expression bound to hnRNPA2B1.	(60)
miRNA	Hepatocyte	RBP: Syncrip/hnRNPQ	Syncrip identifies hEXO (GGCU/A) sequences in target miRNAs through the collaboration of the non-canonical N-terminal RNA recognition region NURR domain and the classical RRM domain.	(61, 62)
miRNA	HEK293T cells	RBP: YBX1	YBX1 interacts with miR-223 through its internal cold shock domain to form hairpin-loop secondary structure, rather than specific recognition motif, which promotes the separation of miR-223 into exosomes.	(63)
miRNA	Pancreatic cancer cells	RBP: SRSF1	SRSF1 mediates enrichment in exosomes of miRNAs with a specific common short motif (e.g., miR-1246) with a motif length of 6 bp and GG bases at positions 3 and 4.	(64)
miRNA	Colon cancer cell	Major vault protein (MVP)	MVP can selectively enrich miR-193a to exosomes and reduce its intracellular content, however, the specific interaction region has not been studied. MiR-193a in turn can affect its target GTPase Rab27B and affect exosome production.	(65, 66)
miRNA	Colon cancer cell	AGO	Ago2 complexes are responsible for the stability of plasma miRNAs, such as miR-16. However, the exact mechanism underlying this interaction remains clear.	(67)
miRNA	Human colonic NCM460 epithelial cells	SP/NK-1R signaling	SP/NK-1R signaling increased the production of exosomes and the level of miR-21 in the exosome cargo.	(68)
miRNA	Human B cells	3’ uridylation/uridinetransferase	MiRNAs with 3’ uridylation were more likely to be secreted into exosomes. This may underlie the mechanism by which cells regulate specific miRNAs functions: either the 3’ uridylation of miRNAs may destabilize RNA, or there is a uridinetransferase in the exocrine (e.g., ZCCHC11 mediates the addition of uracil at the miR-26a terminal).	(69)
miRNA	Prostate cancer cells	3’-end uridylation	Post-transcriptional 3’-end uridylation of miR‐2909 can drive the recruitment of this miRNA for sorting into exosomes.	(70)
circRNA	Platelets	—	Platelets selectively encapsulate shorter size circRNA into exosomes, which may also be related to the RBP binding of circRNA.	(71)
circRNA	HepG2 cells	—	circRNAs with a common 5’-GMWGVWGRAG-3’ motif appear to be more likely to be sorted into exosomes.	(72)
lncRNA	Renal cell carcinoma cell	hnRNPA2B1	There is a special motif at the 5’-end of lncARSR that is able to bind to the RNA-binding protein hnRNPA2B1 and has been sorted into exosomes together with the target miR-198 of IncARSR.	(15)
lncRNA	Human NSCLC cell	hnRNPA2B1	The expression of lncRNA H19 was upregulated in gefitinib-resistance cells of NSCLC. There was also a GGAG substrate in the 5’ terminal region, which was bound to hnRNPA2B1 protein, allowing for its specific sorting into exosomes	(73)
lncRNA	Human breast cancer cell	hnRNPA2B1	In trastuzumab-resistant breast cancer cells, hnRNPA2B1 is overexpressed or silenced, and exosome AGAP2AS1 expression is correspondingly up-regulated or decreased.	(74)
lncRNA	Bladder cancer cell	hnRNPA2B1	lncRNA LNMAT2 specifically binds to hnRNPA2B1 and is packaged into exosomes through its specific sequence of GGAG on the 1930-1960 nt region and the stem-loop structure in this region.	(75)

— No specific molecules were found.

### RNA-Binding Proteins Mediate ncRNAs Sorting Depending on Their Characteristic Motif

It is worth noting that almost all RNAs in cells exists as ribonucleoprotein (RNP) complexes. As such, proteins capable of interacting with RNA (i.e., RBPs) can be critical factors for the promotion of ncRNA transmission in the parent cells and can serve as the intracellular inducers of ncRNA loading in exosomes in the recipient cell (76). It has also been reported that short nucleotide sequences on RNA can guide its transport to different subcellular compartments, including exosomes (77–80).

Proteomic analyses have detected the specific binding of heterogeneous nuclear RNP A2B1 (hnRNPA2B1) to exosomal miR-198 with the RTS motifs. hnRNPA2B1, which is present in exosomes, binds to exosomal miRNA directly and controls its loading into these microvesicles. Moreover, hnRNPA2B1 in exosomes is SUMOylated, and SUMOylation controls the binding of hnRNPA2B1 to miRNAs (77, 79, 81, 82). Another recent study found that epirubicin-treated endothelial cells specifically regulated the extracellular separation of miR-503 by disrupting hnRNPA2B1 (83). In addition to miRNAs, hnRNPA2B1 has been found to specifically regulate lncRNAs. There is a special motif at the 5′ end of lncARSR that can bind to the RBP hnRNPA2B1 and has been sorted into exosomes together with the target miR-198 of lncARSR (15). Lei et al. identified that the expression of lncRNA H19 was upregulated in gefitinib-resistant non-small cell lung cancer (NSCLC) cells and that there was a GGAG substrate in the 5′ terminal region, which was bound to hnRNPA2B1 protein to be specifically sorted into exosomes (73). In trastuzumab-resistant breast cancer cells, hnRNPA2B1 is overexpressed or silenced, and exosome AGAP2AS1 expression is upregulated or downregulated (74). Moreover, lncRNA LNMAT2 specifically binds to hnRNPA2B1 and is packaged into exosomes through its specific sequence of GGAG in the 1930–1960-nt region and the stem-loop structure in this region (75).

Serine and arginine rich splicing factor 1 has been identified as a mediator of exosomal miRNA enrichment in pancreatic cancer cells by binding to a specific miRNA sequence motif (64). Major vault protein can selectively enrich miR-193a to exosomes and reduce its intracellular content; however, this specific interaction region has not been studied. In turn, MiR-193a can affect its target GTPase Rab27B and exosome production (65, 66, 84, 85). Zhang et al. suggested that exosomes package circular RNAs (circRNAs) containing the purine-rich 5′-GMWGVWGRAG-3′ motif, with the characteristic “garbage dumping” and “intercellular signaling” functions (72). Zietzer et al. confirmed that the export of miRNAs into EVs depends on the binding efficiency of the respective miRNAs to hnRNPU. miR-30c-5p, the most significant miRNA regulated by hnRNPU, retains a significant enrichment of the sequence motif AAMRUGCU as a transport signal (56). Wozniak et al. identified a common short sequence of the “AAUGC” motif present in miRNAs that are selectively loaded into exosomes after RILP cleavage, which promotes the movement of MVBs toward the cell periphery and induces selective exosomal miRNA cargo loading. This motif binds the RBP FMR1 and directs miRNA loading into exosomes by interacting with components of the ESCRT pathway (57, 86). Syncrip/hnRNPQ, a highly conserved RBP, can identify hEXO (GGCU/A) sequences in target miRNAs and mediate exosome enrichment through the collaboration of the non-canonical N-terminal RNA recognition region NURR domain and the classical RRM domain (61, 62). Additionally, miR-133 was specifically sorted into H/R-induced EPC-derived exosomes *via* YBX-1 to increase fibroblast angiogenesis and MEndoT (87). Recent discoveries have indicated that circulating Ago2 complexes are responsible for the stability of plasma miRNAs through the KRAS-MEK-ERK signaling pathway, protecting miRNAs contained within EVs from RNase degradation (67, 88, 89). Considering RNA-binding ubiquitin E3 ligase (MEX3C) associates with Ago2 and the adaptor-related protein complex 2 (AP-2), which is involved in miRNA sorting, containing a C-terminal RING finger domain and the hnRNP K homology (KH) domain, miR-451a is specifically sorted into exosomes *via* a ceramide-dependent pathway (90, 91) In general, the concept that specific ncRNA motif binding to RBP is involved in exosomal ncRNA sorting has been confirmed in different types of cells.

### Modification of ncRNAs or RBPs Guide Their Sorting Into Exosomes

Collectively, current research is beginning to uncover new mechanisms by which exosomes are involved in the post-transcriptional modification of ncRNA (92). Reports have shown that miRNAs are modified through a series of processing events after transcription, such as 5′-end phosphorylation, 3′-end adenylation or uridylation, and terminal nucleotide deletion. Khan et al. attempted to develop a method for competence-mass spectrometry, which can be used to perform a multiplex, direct analysis of miRNAs from biological samples, and revealed modifications of miRNAs in serum samples (93). Koppers et al. performed RNA sequencing and bioinformatics analysis and found a non-random distribution of miRNAs between B cells and exosomes. Subsequently, in the urine samples, the 3′-terminal adenylation miRNAs were relatively enriched in the cells, whereas the 3′-end uridine subtypes were found in excess in the exosomes, suggesting that post-transcriptional modifications, especially 3′-end adenylation and uridylation, play the opposite role and may at least partially guide the entry of ncRNAs into exosomes (69). Wani et al. revealed that post-transcriptional modifications, especially 3′-end adenylation and uridylation of miR-2909, exert opposing effects that may contribute to its sorting into exosomes secreted by cancer cells (70).

Emerging evidence suggests that the post-translational modification of RBPs also has a considerable influence on the sorting of specific ncRNAs. Lee et al. showed that cav 1 14 (Y14)-tyrosine phosphorylation leads to interactions between caveolin 1 (cav 1) and hnRNPA2B1. Cav 1, as a lipid-raft scaffolding protein, has been proposed to induce local membrane composition and curvature, constitutes a complex with HNRNPA2B1-miRNAs, subsequently directs theirs their routing towards exosomes in lung epithelial cells. Oxidative stress induces the O-GlcNAcylation of hnRNPA2B1, resulting in a robustly altered hnRNPA2B1-bound miRNA repertoire. Notably, cav-1 pY14 also promoted hnRNPA2B1 O-GlcNAcylation. Functionally, macrophages serve as the principal recipients of epithelial EVs in the lungs. EV-containing cav-1/hnRNPA2B1 complex-bound miR-17/93 activates tissue macrophages (60). Furthermore, hnRNPA2B1 in exosomes is SUMOylated, which controls the binding of hnRNPA2B1 to miRNAs (58). The KRAS-MEK-ERK pathway-dependent phosphorylation of Ago2 has been demonstrated to exert specific control over the sorting of let-7a, miR-100, and miR-320a into exosomes (94).

### Content of Intracellular Associated RNAs Modulates the Sorting of ncRNAs Into Exosomes

It has been reported that ncRNAs can be modulated by their upstream or downstream RNAs, thus being packaged into exosomes either *via* passive leakage or in an active secretion manner. The most classical is the RNA processing of miRNA, which affects miRNA enrichment in exosomes. It is known that miRNA maturation in the cytoplasm requires a type II endoribonuclease known as the Dicer enzyme. It cleaves the stem loop structure of pre-miRNA and produces ~22-nt miRNA double strands, which are unwound into mature single-stranded miRNAs and combined with RNA-induced silencing complex (RISC). The co-localization and accumulation or relocalization of miRISC components in MVBs is thought to facilitate the sorting of miRNAs into exosomes (95, 96).

It has been proven that ncRNAs can interact with their target RNAs to regulate the content of their own or target RNAs in cells, after which they are sorted into exosomes passively or actively. Squadrito et al. suggested that the occurrence of physiological (cell-activation-dependent) or artificial overexpression of miRNA target sequences (mRNA) in macrophages contributes to the enrichment of the corresponding miRNA exosomes and P-bodies. Perhaps, through such a mechanism, the miRNA loading in the producer cells can be reduced; however, through this mechanism, the activity of miRNA can be affected and cell homeostasis can be successfully achieved (97). In renal cell cancer cells, Qu et al. found a new lncRNA, lncARSR, which has a specific GGAG/CCCU at its 5′-end and can bind to the RBP hnRNPA2B1 and is selected as an exosome. Interestingly, the target of lncARSR, miR-198, rather than the target miR-18a, can be secreted into the exosomes along with the lncARSR-hnRNPA2B1 complex (15, 58). In HEK293T and MCF-7 cells, miR-7, the target of CDR1as, was artificially upregulated. Subsequently, Li et al. observed that CDR1as circRNA levels in exosomes were significantly downregulated. This result indicates that the sorting of circRNA to exosomes may be regulated by the level of related miRNAs in the production cells and may transfer biological activity to the recipient cells (98).

### Secondary/Tertiary Structure of ncRNAs Regulates Their Sorting

These interactions could occur because the secondary/tertiary structures of ncRNAs, as opposed to those of other nucleic acid sequences, partially result in a greater protein binding capacity. Shurtleff et al. discovered that RBP Y-box protein I (YBX1) binds to and is required for the sorting of miR-223 in HEK293T cells. YBX1 interacts with miR-223 through its internal cold shock domain to form a hairpin-loop secondary structure, rather than a specific recognition motif, which promotes the separation of miR-223 into exosomes (63). Platelets are rich in circRNAs and seem to be more inclined to secrete smaller-sized circRNAs into exosomes than larger ones. It is reasonable to speculate that the mechanism by which circRNAs are sorted into exosomes is related to their circular tertiary structure, which needs to be further characterized (71).

## Clinical Application of Exosomal ncRNAs

### Biological Effects of Exosomal ncRNAs

**Figure 2:**
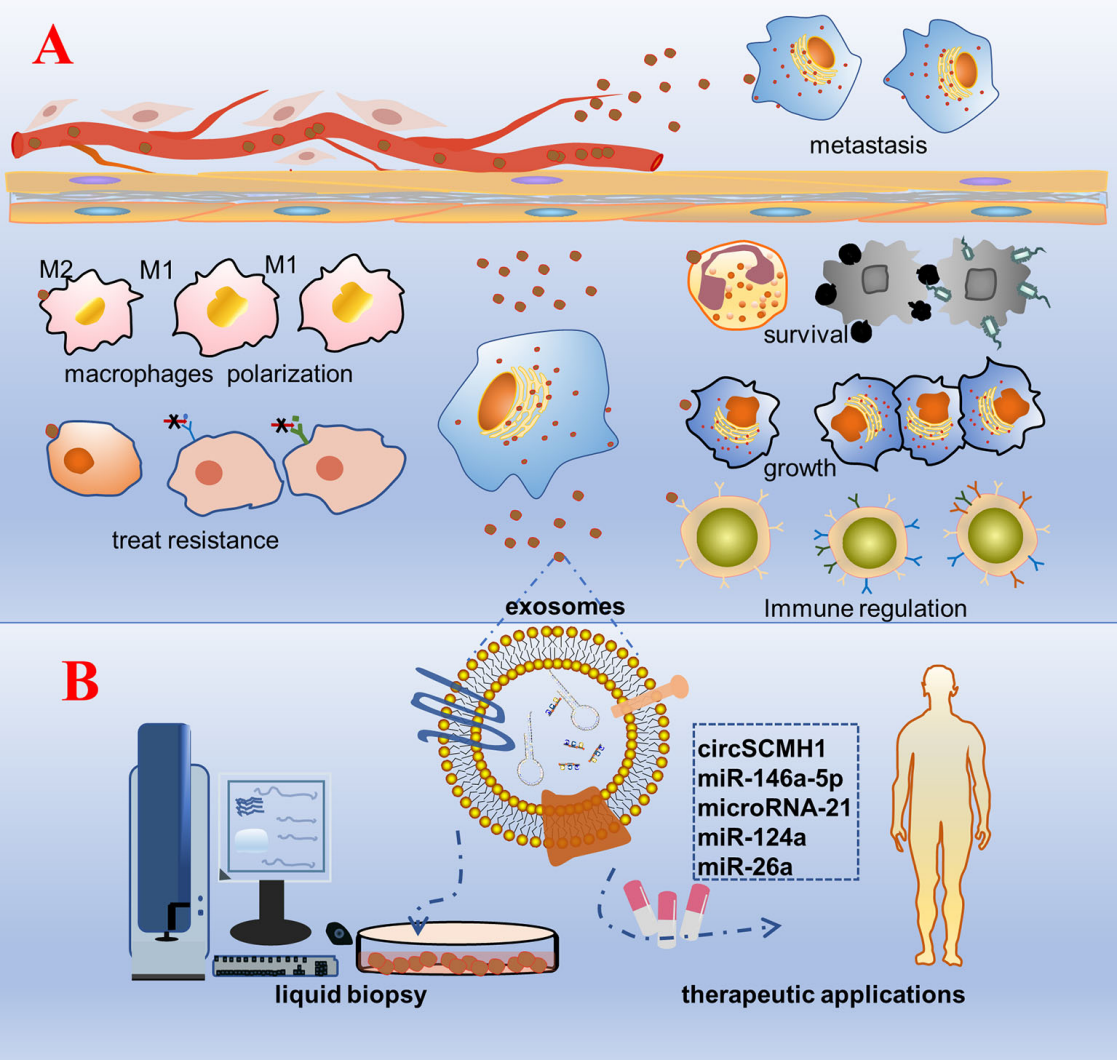
Schematic diagram of the biological function and clinical application prospects of exosomal non-coding RNAs. **(A)** Biological function: cells are stimulated by factors, such as tumorigenesis, secrete exosomes wrapped with bioactive non-coding RNAs, and are accepted by the recipient cell. As a result, a series of phenotypic changes occur: pathogenic microorganisms escape the body’s immune surveillance to survive; growth of specific recipient cells; regulation of the number and functions of immune cells, such as T cells and NK cells; polarization of macrophages and the inflammatory response; change in the tolerance of the recipient cells to treatment; transmission to distant tissue cells through body fluids inducing cancer metastasis. **(B)** Clinical application: purification, separation, and detection of exosomes in various body fluids, construction of a platform for rapid detection and analysis of diseases, and engineering of exosomal non-coding RNAs that are promising for treatment.

Exosomes are known to be rich in biological information (proteins, nucleic acids, etc.). They are not only acting as tissue sampling to reflect the state of parent cells, but also representative tools to transmit cell-to-cell communication information. Exosomes play a vital role in mediating cell–cell communication and transporting cargo from donor cells to recipient cells, regardless of whether the recipient cells are located in distant or nearby tissues (7, 99). Bidirectional cell–cell communication involving exosome-borne cargos, such as ncRNAs, has emerged as a critical mechanism. As natural intercellular shuttles of ncRNAs, exosomes influence an array of developmental, physiological, and pathological processes in the recipient cell or tissue to which they can be selectively targeted (**Figure 2**) (100). For example, Exosomal ncRNAs could interact with many inflammatory factors and inflammatory cells to influence the progression of inflammatory diseases. The exosomes promoted macrophage M1 differentiation at least partially *via* transferring pro-inflammatory miRNAs, such as miR-155. Moreover, exosome-mediated miR-155 inhibitor delivery significantly prevented DSS-induced colitis (101). Hepatocyte exosomes induced by lipotoxic injury are rich in miR-192-5p. It can regulate the Rictor/Akt/FoxO1 signaling pathway, induce an increase in the expression of M1 macrophages and inflammatory factors, and affect the progression of steatohepatitis (102). Exosomes from patients with septic shock convey miRNAs and mRNAs related to inflammatory response for intercellular communication (103). In general, exosomal ncRNAs are essential as a major contributor that regulates delivery and reduces inflammatory response (59). The ncRNAs content of exosomes could be regulated by the physiological state of cells and may play a role in maintaining tissue homeostasis and synchronizing the functional state of cells. For example, miRNAs transferred from mother’s milk to the infant may play a crucial role in the development of the infant’s immune system (104). Exosomal miRNAs have been proven to act as regulators of neuron- astrocyte crosstalk, osteoblast differentiation, myoblast differentiation, and so on (105–108). Exosomal ncRNAs play a key role in premetastatic niche formation and metastasis (109, 110). Zhang et al. observed that the expression of circSATB2 in serum exosomes of patients with NSCLC was higher than that in non-cancerous donors, which was related to lymph node metastasis of lung cancer and could promote the proliferation of normal bronchial epithelial cells. This suggests that exosomal circSATB2 can transmit cell communication and promote the metastasis of tumor cells (111). Exosomes imposed by pathogenic microorganisms, such as viruses, bacteria, and parasites, may be exploited for the superior delivery of ncRNAs to evade host immune surveillance *in vivo* (112–114). *Schistosoma japonicum* egg-derived exosomal Sja-miR-71a attenuated pathological progression and liver fibrosis in *S. japonicum* infection (115). Adult *Schistosoma* secretes exosomal miRNAs that are internalized by Th cells to evade immune surveillance (116). Exosomes are soluble biological mediators obtained from mesenchymal stem cells (MSCs) cultured *in vitro*. MSC-derived exosomes produced under physiological or pathological conditions are central mediators of intercellular communication by transporting proteins, lipids, mRNAs, siRNA, rRNAs, and ncRNAs to neighboring or distant cells.

### Exosomal ncRNA as a Specific Biomarker for Liquid Biopsy

Liquid biopsy is a minimally invasive method for analyzing solid tissues, blood, and other body fluids. Exosomes can be conveniently detected in almost all human body fluids, making them an ideal indicator for liquid biopsy. Exosomes derived from different cell types and statuses have been shown to possess distinct RNA profiles, particularly ncRNAs. These new analytes represent an alternative tool to complement the diagnosis, monitoring, and prediction of response to treatment of tumor processes, as well as other human disease processes, such as those in viral and parasitic infections (117, 118). ncRNAs are important regulators of cellular signaling that can be detected and released into circulation with high stability *via* packing in exosomes (119–122). Therefore, the use of exosomal ncRNAs has promising prospects in liquid biopsy in case of diseases and may continue to be an exciting focus of research in the field.

Owing to the rapid development of various exosome detection technologies in recent years, exosomal ncRNAs have become a specific and effective biomarker for clinical liquid biopsy (123–125). Methods of exosome isolation to date include ultracentrifugation (mostly approved for exosome purity), ultrafiltration, size-exclusion chromatography, polymer precipitation, immunoaffinity chromatography, and microfluidics-based techniques (126–128). Wang et al. described the construction and testing of an electrochemical biosensor for the sensitive detection of exosomal miRNAs. The electrochemical biosensor exhibited good selectivity for miR-21 detection; showed benefits of simple operation, low cost, and portability; and provided a promising platform for the early diagnosis and screening of tumor biomarkers and the development of devices for point-of-care testing (129). Wu et al. established a platform for the simultaneous multiplex analysis of multiple exosome biomarkers (such as proteins and miRNAs) in clinical biological fluids, which not only allows for the observation of the tissue status of biomarkers in clinical samples but also shows that exosome subsets can more accurately distinguish the prognosis of patients (130). Zabegina et al. isolated exosomes with thyroid-specific surface molecules by immunobeads followed by miRNA analysis, demonstrating possibly improved diagnostic potency (130). Serum exosomal miRNA profiles of steroid-induced osteonecrosis of the femoral head (SONFH) and hsa-miR-135b-5p may be a unique diagnostic biomarker for SONFH (131). Zheng et al. found that the expression level of exosomal lnc-SLC2A12-10:1 was significantly correlated with tumor size, TNM stage, lymph node metastasis, and degree of differentiation, suggesting that exosomal lnc-SLC2A12-10:1 may be a potential noninvasive biomarker for the diagnosis and prognosis monitoring of gastric cancer (132). In summary, ncRNAs derived from exosomes are considered potential new biomarkers for various diseases, especially cancer, and can be easily detected in liquid biopsies.

### Therapeutic Applications of Engineered Exosomal ncRNAs

A variety of ncRNA molecules are known to function in human diseases. However, safety issues with delivery systems have limited the exploration of the potential therapeutic roles of ncRNAs. Engineered EVs carrying therapeutic molecules are promising candidates for disease therapy. In recent years, exosomes have been discovered with low immunogenicity, positive safety in clinical trials, and the ability of selectively homing to inflammation and tumor sites. Therefore, engineered exosomal ncRNAs have great therapeutic potential (133–137).

Yang et al. found that in an ischemic stroke model, the engineered extracellular vesicular rabies virus glycoprotein-circSCMH1 selectively transmits circSCMH1 to the brain and mechanically binds to the transcription factor MeCP2, thus resulting in the inhibition of MeCP2 target gene transcription and promoting the functional recovery of stroke in animals (138). Exosomal miRNAs and proteins isolated from hiPSC-NSC cultures have many functions, such as neuroprotection and anti-inflammation. The intranasal administration of exosomes can be absorbed by a variety of nerve cells, which is beneficial for brain repair after injury or disease (139). A separate study showed that secreted exosomes coated with miR-146a-5p from MSCs relieved Group 2 innate lymphoid cells (ILC2s) in innate airway inflammation, showing significant advantages of low immunogenicity and high biosafety (140). Engineered exosomes loaded with anti-inflammatory agents, such as miRNA-21, could be used to target macrophages in the inflammatory region to regulate inflammatory responses for achieving the ability to regulate inflammatory responses when needed (141). Engineered exosomal ncRNAs have also shown great advantages in the treatment of tumors. In addition to the unique properties of MSCs, MSC-derived exosomal ncRNAs exert desirable therapeutic effects (142). Lang et al. reported that bone marrow-derived MSCs could encapsulate miRNAs, such as miR-124a, into exosomes, and these engineered exosomes could be used to treat mice harboring intracranial glioma stem cell. It was found that engineered exosomes could systematically transmit anti-glioma miRNAs to glioblastomas for longer survival (143). Liang et al. introduced a new approach for the targeted delivery of exosomes loaded with functional miR-26a to scavenger receptor class B type 1-expressing liver cancer cells, resulting in decreased rates of cell migration and proliferation (144).

## Conclusion

Nanoscale exosomes encapsulating a variety of cargos, including ncRNAs, which protect the cargo from degradation by various enzymes in the extracellular space, are vital for intracellular and intercellular communication. Exosomes can be selectively secreted *via* ESCRT-dependent or ESCRT-independent pathways. Exosomes have multiple functions in physiological and pathological processes, and ncRNAs can also play an important regulatory role in these processes. The relationship between them has attracted great research interest in recent years. Subsequently, an increasing number of studies have revealed that exosomal ncRNAs are involved in pathogenic microbial infection and inflammatory disease, tumor invasion and metastasis, immunoregulation and immunotherapy, and resistance to treatment. However, how ncRNAs in exosomes are selectively packaged and then transported to target cells remains unclear, which hinders the prospects of clinical applications of exosomal ncRNAs as biomarkers. Elucidating the mechanism by which cells sort specific ncRNAs into the circulation will aid in the selection of more representative exosomal RNAs and pave the way for advances in the early diagnosis of diseases. Exosomal ncRNAs can transmit signals, such as those involved in growth promotion, invasion, metastasis, and angiogenesis. Moreover, animal experiments have confirmed their effect on the disease phenotype. Thus, we believe that understanding how these disease-causing or disease-suppressing exosomal ncRNAs are sorted, secreted, and spread to adjacent or distant cells in the initial stages of a disease will underlie the development of timely, accurate, and effective early intervention measures.

Although plenty of methods have been developed to isolate exosomes, it has been found that extracellular ncRNAs could be mixed with a variety of exosomal vectors such as lipoprotein and Argonaute protein, which obviously interferes with the accuracy of studies on exosomal ncRNAs. Therefore, researchers are making unremitting efforts to improve the technology of exosome purification and separation. In addition, exosomes have been recognized as a class of aggregated nanoparticles with different properties of extracellular vesicles. Currently, there is a lack of specific characterization of the heterogeneity of different exosome subtypes. Significant differences of the proteins, lipids, and nucleic acids contained in different exosome subtypes have been demonstrated. The heterogeneity of secreted nanoparticles is increasingly recognized. Numerous studies have exploited new technologies to characterize EV subtypes. Ayuko Hoshino and his team deconvolved the heterogeneity of extracellular nanoparticles and defined three distinct subsets: small exosomes (Exo-S, 50–70 nm), large exosomes (Exo-L, 90–120 nm) and exomeres. The exomeres obtained through sequential ultracentrifugation (SUC) were non-membranous particles (<50 nm). Many of the most abundant miRNAs are more associated with extracellular exomeres than parent cells or Exo-S components. Furthermore, some RBPs proved to be related to the mechanism of sorting ncRNA, such as YBX1 and MVP protein, were also strongly correlated with exomeres components. Therefore, there is an urgent need to identify signature proteins that can more effectively distinguish EV subtypes (145–149). Therefore, the application of the enriched ncRNAs in different exosome subtypes in liquid biopsy or clinical treatment also needs further study.

Technological advances have enabled researchers to opt for more effective methods for the separation of high-purity exosomes, allowing for the in-depth study of the mechanism underlying the non-random distribution of functional molecules to exosomes in cells. It is reasonable to speculate that hundreds of millions of patients, especially those with poor treatment outcomes, will greatly benefit from the in-depth study of the mechanism and role of exosomal ncRNAs.

## Author Contributions

YQ and MW conceived, wrote, and edited the manuscript. YQ and PL designed figures and wrote and edited the manuscript. MW and ZZ wrote and edited the manuscript. All authors contributed to the article and approved the submitted version.

## Funding

This work is supported by grants from the National Science Foundation for Young Scientists of China (Grant 81802871); Graduate Scientific Research and Innovation Project of Central South University (No. 1053320192778).

## Conflict of Interest

The authors declare that the research was conducted in the absence of any commercial or financial relationships that could be construed as a potential conflict of interest.
