# Corrigendum: Insights Into Exosomal Non-Coding RNAs Sorting Mechanism and Clinical Application

**DOI:** 10.3389/fonc.2021.781714

**Published:** 2021-10-11

**Authors:** Yi Qiu, Peiyao Li, Zuping Zhang, Minghua Wu

**Affiliations:** ^1^Hunan Cancer Hospital and the Affiliated Cancer Hospital of Xiangya School of Medicine, Central South University, Changsha, China; ^2^Cancer Research Institute, School of Basic Medical Science, Central South University, Changsha, China; ^3^Key Laboratory of Carcinogenesis and Cancer Invasion of Ministry of Education, China National Health Commission Key Laboratory of Carcinogenesis, Xiangya Hospital, Central South University, Changsha, China

**Keywords:** exosome, extracellular vesicles, non-coding RNAs, sorting mechanism, cell-cell communication, ncRNAs

In the published article, there was an error in **Affiliation 1**. Instead of “Hunan Provincial Tumor Hospital and the Affiliated Tumor Hospital of Xiangya Medical School, Central South University, Changsha, China”, it should be “Hunan Cancer Hospital and the Affiliated Cancer Hospital of Xiangya School of Medicine, Central South University, Changsha, China”.

The authors apologize for this error and state that this does not change the scientific conclusions of the article in any way. The original article has been updated.

## Publisher’s Note

All claims expressed in this article are solely those of the authors and do not necessarily represent those of their affiliated organizations, or those of the publisher, the editors and the reviewers. Any product that may be evaluated in this article, or claim that may be made by its manufacturer, is not guaranteed or endorsed by the publisher.

